# Severe Eosinophilic Meningoencephalitis Secondary to Suspected Neuroangiostrongyliasis with a Good Clinical Outcome

**DOI:** 10.1155/2019/4037196

**Published:** 2019-05-26

**Authors:** Fabian Chiong, Andrew R. Lloyd, Jeffrey J. Post

**Affiliations:** ^1^Department of Infectious Diseases, Prince of Wales Hospital, Sydney, NSW, Australia; ^2^Prince of Wales Clinical School, University of New South Wales, Sydney, NSW, Australia; ^3^Kirby Institute, University of New South Wales, Sydney, NSW, Australia

## Abstract

*Angiostrongylus cantonensis* has caused sporadic cases of eosinophilic meningoencephalitis in Sydney, Australia. We describe a 36-year-old man who presented subacutely with fevers, reduced level of consciousness, confusion, ophthalmoplegia, and urinary incontinence. He was diagnosed with severe eosinophilic meningoencephalitis secondary to suspected *Angiostrongylus cantonensis* based on clinical, serological, and radiological findings. The patient was treated with albendazole and prednisolone with full neurological recovery. Management of neuroangiostrongyliasis with anthelminthic is controversial as it is thought to cause worsened outcomes through inciting an inflammatory response as a result of parasite killing. We managed to successfully treat our patient using albendazole and prednisolone and achieved a good outcome.

## 1. Introduction

We describe an unusual case of severe suspected *Angiostrongylus* meningoencephalitis with a subacute presentation of neurological symptoms over months. The MRI brain was impressive showing punctate and linear haemorrhagic tracks which were indicative of helminth invasion. The patient responded well to treatment and survived without any neurological impairment with a diagnosis which is normally associated with a very high mortality and morbidity rate.

## 2. Case Presentation

A 36-year-old homeless man was brought to hospital by concerned citizens due to drowsiness. A history was not able to be obtained from him as he had become mute. On examination, he had a Glasgow Coma Scale (GCS) of 9—eye movement 3, verbal response 1, and motor response 5. He was febrile (38.5°C), tachycardic (HR 115 bpm) with normal blood pressure, and normal oxygen saturation and respiratory rate. He was found to have injection marks on his arms and forearms, suggesting that he was an intravenous drug user. He was incontinent of urine and had reduced lateral gaze of the right eye with dysconjugate eye movements. Primitive reflexes including glabellar tap and the rooting reflex were present. Other neurological examination findings were limited due to poor patient cooperation, but no other clear neurological signs were elicited.

**Figure 1:**
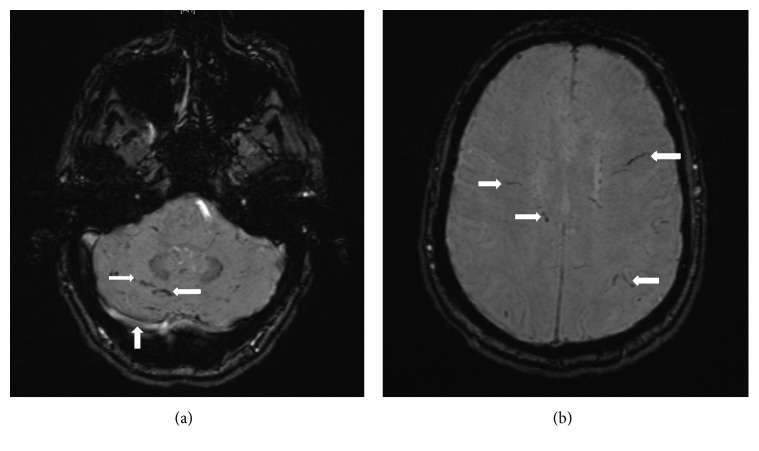
Magnetic resonance imaging of the brain—susceptibility-weighted imaging maximum intensity projection (SWI mip) sequence showing linear and punctate haemorrhagic lesions throughout the cerebrum and cerebellum and leptomeningeal enhancement in the right cerebellum folia.

**Figure 2:**
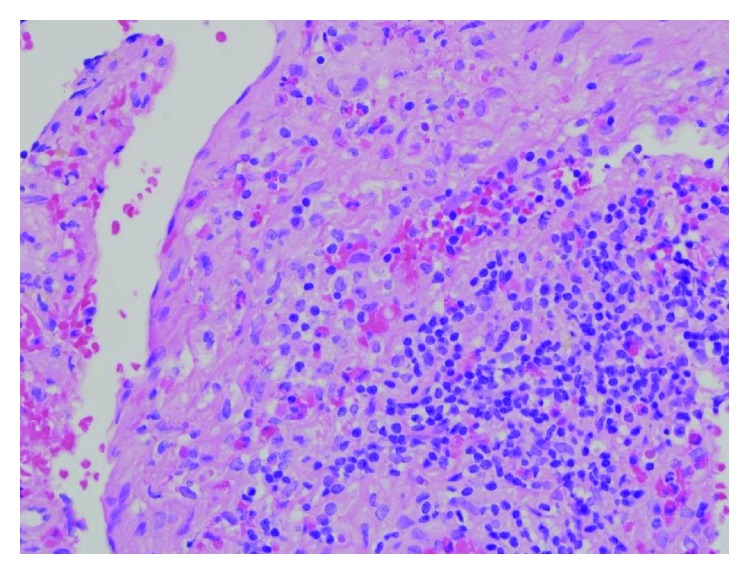
Histopathology from brain biopsy revealing eosinophilic infiltration in brain parenchyma and meninges.

Urgent investigations were performed which revealed a peripheral blood leukocytosis with an eosinophilia (3.34 × 10^9^/L, reference interval (RI) 0.04–0.44 × 10^9^/L). Renal function was normal, and liver function tests were mildly deranged with a mixed obstructive and hepatitic picture. He was tested and found to have chronic hepatitis C virus infection, but was negative for human immunodeficiency virus and hepatitis B virus infections. A lumbar puncture revealed intracranial hypertension with an opening pressure of 25 cm H_2_O (RI 5–15 cm H_2_O). There was a cerebrospinal fluid (CSF) pleocytosis (465 × 10^6^/L white blood cells), with predominantly polymorphonuclear cells (85%) and 516 × 10^6^/L red blood cells. The CSF protein was mildly elevated (1.12 g/L [RI] 0.15–0.45 g/L), and the glucose was low (2.3 mmol/L [RI] 2.5–5.5 mmol/L). He was treated with empirical antibacterial and antiviral therapy for meningoencephalitis. CSF bacterial culture, India ink stain, cryptococcal antigen, flow cytometry, and polymerase chain reaction (PCR) for herpes simplex virus, varicella zoster virus, and enteroviruses were all negative. Initial computed tomography (CT) of the brain was unremarkable, and magnetic resonance imaging of the brain (MRIB) did not reveal any abnormalities; however, the quality of the images was degraded due to motion artefact. He deteriorated further in his conscious state to the extent that he was comatose with involuntary shaking of limbs. He was intubated and transferred to intensive care unit for further care. An electroencephalogram did not show any epileptiform activity. Giemsa stain on CSF was requested and revealed that a third of the polymorphonuclear cells were eosinophils. Repeat MRIB (while the patient was comatose and intubated) revealed linear and punctate haemorrhagic lesions throughout the cerebrum and cerebellum on susceptibility-weighted images maximum intensity projection (SWI mip) sequence (Figures 1(a) and 1(b)). These findings were consistent with helminth migration. There was also leptomeningeal enhancement in the right cerebellar folia (Figure 1(a)) and evidence of widespread meningitis on MRIB. A brain biopsy was performed which revealed eosinophilic meningoencephalitis without detection of helminths, vasculitis, or malignancy (Figure 2). A CT chest was also performed which revealed bibasal ground-glass changes, potentially consistent with helminthic migration. Bronchial alveolar washings obtained via bronchoscopy revealed eosinophil accumulation but did not reveal any helminthic larvae.

The patient was started on therapy for suspected *Angiostrongylus cantonensis* meningoencephalitis with albendazole (15 mg/kg daily) and prednisolone (50 mg daily) orally. His GCS improved significantly, but he complained of worsening headache within the first 48 hours of treatment. After 3 days of albendazole and prednisolone treatment, the headache improved markedly and he was fully alert and communicating coherently. Further history was sought and revealed that he had headache, fevers, agitation, unsteady gait, and occasional urinary incontinence for a few months prior to hospital presentation. He denied any slug ingestion or recent travel history. There were no residual neurological deficits, and the peripheral eosinophilia resolved promptly after commencement of treatment. On serological testing, *Angiostrongylus cantonensis* IgG antibodies were positive in the cerebrospinal fluid (2.52 IU, RI 1.0) and serum (3.99 IU, RI 1.0). *Cysticerca* IgG was also positive in the serum and CSF; however, the confirmatory immunoblot test against *Cysticerca* was negative, and the clinical and radiological findings were more consistent with angiostrongyliasis. The source of this *Angiostrongylus* infection remained unclear. He discharged against medical advice after 8 days in hospital, and he was provided another 2 weeks' worth of albendazole and a weaning course of prednisolone. He was offered outpatient follow-up appointments, but he failed to attend. He was next clinically assessed seven months later and stated that he had been adherent to the prescribed medications after discharge. He had no apparent complications from the suspected neuroangiostrongyliasis.

## 3. Discussion

The nematode *Angiostrongylus cantonensis,* also known as rat lung worm, is one of the major causes of eosinophilic meningitis and meningoencephalitis. It was first discovered in the pulmonary arteries of rats in Guangzhou (Canton), China, in 1935 [1]. It was first reported to cause human infection in Taiwan in 1945 [2]. It is now found in many parts of the world and endemic in temperate parts of Southeast Asia, Eastern Asia, Pacific Islands, Central America, South America, and the Caribbean [3, 4]. There have been sporadic cases of *Angiostrongylus cantonensis* infection in humans in the eastern coast of Australia since 1971 [5–8]. It was first reported in Sydney in 2001 [5]. The previously reported Australian cases of *Angiostrongylus* meningoencephalitis either died or experienced significant long-term neurological impairment [7, 8]. This is a unique reported case of severe *Angiostrongylus* meningoencephalitis with significant neurological deficits in which there was a full recovery.

Rats are definitive hosts to the worm, *Angiostrongylus cantonensis*. The female form of the worm lay eggs in the pulmonary arteries of the rats, and they mature into first stage larvae (L1). The L1 migrate to the pharynx, are swallowed, and excreted in rat faeces. Snails or slugs which act as intermediate host feed on rat faeces and enable the larvae develop into second (L2) and then third stage larvae (L3) [3]. Human angiostrongyliasis occurs when stage three larvae (L3) of the nematode are ingested either unintentionally (food contaminated by molluscs' slime) or by consumption of raw snails or slugs. The US Centers for Disease Control and Prevention (CDCP) published a report which identified fifty-five percent of twelve angiostrongyliasis cases were likely due to consumption of raw vegetables [9]. Our patient did not have a history of raw seafood, mollusc, or snail ingestion, and the mechanism of infection is unclear. The ingestion of L3 was most likely inadvertent from contaminated food.

The worm migrates to the central nervous system in humans and then dies inciting an inflammatory response. The tissue damage in the central nervous system is due to both the helminth migration and inflammatory response, leading to a range of neurological symptoms—most commonly headache, neck stiffness, fever, vomiting, back pain, and paraesthesia [3]. The incubation period of *Angiostrongylus cantonensis* is highly variable from days to months depending on the inoculum [10, 11]. The inoculum seems to be inversely proportionate to the acuity of the onset of the disease. The subacute progressive neurological presentation with behavioural change over a few months was unique in this case and may suggest that the inoculum ingested was small. The median incubation period of *Angiostrongylus cantonensis* infection in human is 11 days following ingestion of infective larvae [4].

We managed to establish that our patient had eosinophilic meningoencephalitis promptly due to the presence of clinical features of meningoencephalitis, peripheral eosinophilia, and demonstration of eosinophilic pleocytosis in CSF. However, determining the underlying aetiology of eosinophilic meningoencephalitis was challenging. The brain biopsy and initial imaging assisted us to exclude vasculitis and malignancy such as lymphoma. We found that the MRI brain with SWI protocol was useful by demonstrating linear and punctate haemorrhagic lesions throughout the cerebrum and cerebellum suggestive of helminth migratory tracts causing microcavities. The initial CTB and MRIB without the SWI protocol failed to demonstrate those changes to aid in the diagnosis. The MRI findings in this case were similar to the pathologically confirmed cases seen in Sydney previously [7]. Parasitologically confirmed cases of *Angiostrongylus cantonensis* in CSF or brain biopsy are rare [3, 12]. Occasionally, the helminthic infection is diagnosed postmortem [7]. Thus, indirect evidence to detect the presence of the parasites is crucial. In addition to imaging, detecting an immunological response to the parasites is helpful in diagnosing angiostrongyliasis. The enzyme-linked immunosorbent assay (ELISA) testing used in this case employed a crude extract of *Angiostrongylus cantonensis* antigens prepared from larvae. This ELISA technique is highly sensitive but not specific in diagnosing angiostrongyliasis. The serological testing done on this patient was positive for both *Angiostrongylus* and *Cysticerca* species. This indicates that serological testing can cross react between different species of parasites. However, the immunoblot assay for *Cysticerca* was negative, and the radiological findings were not consistent with cysticercosis which helped us to conclude that the diagnosis was *Angiostrongylus* meningoencephalitis. Residual serum for a confirmatory western blot analysis for *Angiostrongylus* was not available.

Optimal management of *Angiostrongylus* meningoencephalitis is uncertain, especially in patients with severe neurological deficits. CDCP guidelines recommend supportive treatment with analgesics, careful removal of CSF to relieve headache as a result of increased intracranial pressure, and corticosteroids to dampen inflammation [13]. Anthelminthics such as albendazole are thought to incite an inflammatory response due to dying organisms with potential worsening of neurological deficits [13, 14]. Albendazole was also thought not to be beneficial in addition to corticosteroids in an observational study [15]. On the other hand, albendazole has been widely used in Thailand, China, and Taiwan with favourable effects in reducing the duration of disease, relieving symptoms, and aiding in full recovery [3, 10]. Albendazole was shown to shorten the duration of headache in eosinophilic meningitis in a prospective, randomised, double-blinded, controlled study with a sample size of 71 conducted in Thailand [16]. Consistent with this evidence, we used albendazole and high-dose prednisolone in our patient with good effect. Albendazole is a relatively safe medication with main side effect of headache (likely due to inflammatory reaction to dying parasites), hepatotoxicity, and occasional gastrointestinal side effects of nausea and vomiting [17].

Uncomplicated eosinophilic meningitis is usually self-limiting and has a good prognosis [3]. In contrast, the prognosis of severe eosinophilic meningoencephalitis typically remains guarded. Mortality rates were more than 90% for patients who became comatose with severe eosinophilic meningoencephalitis due to *Angiostrongylus cantonensis* infection [18]. A complete neurological recovery after severe neurological impairment from neuroangiostrongyliasis appears to be uncommon. We theorise that our patient recovered fully as a result of the combination of a small inoculum of infective larvae and prompt combination treatment with albendazole and prednisolone.

This case highlights the need to consider *Angiostrongylus cantonensis* as a cause of eosinophilic meningoencephalitis and that a good clinical response to treatment can be achieved.
